# Delineating Neural Structures of Developmental Human Brains with Diffusion Tensor Imaging

**DOI:** 10.1100/tsw.2010.21

**Published:** 2010-01-21

**Authors:** Hao Huang

**Affiliations:** Advanced Imaging Research Center, University of Texas Southwestern Medical Center, Dallas, TX, USA

**Keywords:** DTI, fetal brain, atlas, tractography, 3D, microstructure

## Abstract

The human brain anatomy is characterized by dramatic structural changes during fetal development. It is extraordinarily complex and yet its origin is a simple tubular structure. Revealing detailed anatomy at different stages of brain development not only aids in understanding this highly ordered process, but also provides clues to detect abnormalities caused by genetic or environmental factors. However, anatomical studies of human brain development during the fetal period are surprisingly scarce and histology-based atlases have become available only recently. Diffusion tensor imaging (DTI) measures water diffusion to delineate the underlying neural structures. The high contrasts derived from DTI can be used to establish the brain atlas. With DTI tractography, coherent neural structures, such as white matter tracts, can be three-dimensionally reconstructed. The primary eigenvector of the diffusion tensor can be further explored to characterize microstructures in the cerebral wall of the developmental brains. In this mini-review, the application of DTI in order to reveal the structures of developmental fetal brains has been reviewed in the above-mentioned aspects. The fetal brain DTI provides a unique insight for delineating the neural structures in both macroscopic and microscopic levels. The resultant DTI database will provide structural guidance for the developmental study of human fetal brains in basic neuroscience, and reference standards for diagnostic radiology of premature newborns.

## INTRODUCTION

### Brief Principles of DTI

Diffusion tensor imaging (DTI)[1] is an imaging technique of magnetic resonance imaging (MRI). It comes from the measurements of water diffusion. Modern MR scanners are usually equipped with three orthogonal gradient systems. These gradients have different applications. Besides being used mostly as "imaging gradients", one application of these gradients is to be used as "diffusion gradients". A typical diffusion sequence[2] is featured with a pair of diffusion gradients placed on either side of the refocusing pulse. By sampling the diffusion orientations in the three-dimensional space, the diffusion profile can be measured with signals from a diffusion weighted image (DWI) correspondent to a certain diffusion orientation. Since a diffusion tensor has six degrees of freedom, at least six diffusion-sensitized images or DWIs need to be acquired to fit the tensor besides a nondiffusion image. The properties of the three-dimensional ellipsoid are usually defined by the following parameters: the length of the longest, middle, and shortest axes (called eigenvalues $\lambda_1$, $\lambda_2$, and $\lambda_3$) and their orientations (called eigenvectors $\mathbf{v_1}$, $\mathbf{v_2}$, and $\mathbf{v_3}$). As the three axes are orthogonal to each other, only six parameters, i.e., $\lambda_1$, $\lambda_2$, $\lambda_3$ and x, y, z component of $\mathbf{v_1}$, are independent.

DTI-derived images can be obtained from the eigenvalues and eigenvectors of the diffusion tensor. Three DTI-derived images are widely used, namely anisotropy map[3,4,5,6,7], directionally encoded color map[8,9], and apparent diffusion coefficient (ADC) map. With these DTI-derived contrasts, detailed neural structures have been revealed in human (e.g., [4,8,10,11,12,13,14,15]) and mouse brains(e.g., [16,17,18,19]). How these DTI-derived metrics are generated is described below.

Fractional anisotropy (FA)[20] is probably used mostly to characterize the anisotropy of the tensor. The equation of FA is as follows:

$$FA = \frac{\sqrt{(\lambda_1-\lambda_2)^2+(\lambda_1-\lambda_3)^2+(\lambda_2-\lambda_3)^2}}{\sqrt{2}\sqrt{\lambda_1^2+\lambda_2^2+\lambda_3^2}} \tag{1}$$

The values of FA range from 0 to 1. The higher the value of FA, the more elongated the shape of the diffusion ellipsoid looks. ADC is a physical value and usually has the unit of $10^{-3}$ $mm^2$/sec. It can be obtained with the simple average of the three eigenvalues:

$$ADC = (\lambda_1+\lambda_2+\lambda_3)/3 \tag{2}$$

In addition, the eigenvector associated with the largest eigenvalue ($\mathbf{v_1}$), or primary eigenvector, can be used as an indicator of fiber orientation. A DTI directionally encoded color map is usually generated to combine the information of FA and $\mathbf{v_1}$. For the color presentation, 24-bit color is used, in which each RGB color has 8-bit (0-255) intensity levels. The unit vector $\mathbf{v_1}$ (= [$\mathbf{v_{1x}}$, $\mathbf{v_{1y}}$, $\mathbf{v_{1z}}$]) always fulfills the condition: $\mathbf{v_{1x}}^2 + \mathbf{v_{1y}}^2 + \mathbf{v_{1z}}^2 = 1$. Intensity values of $\mathbf{v_{1x}}^2$*255, $\mathbf{v_{1y}}^2$*255, and $\mathbf{v_{1z}}^2$*255 will be assigned to the R(ed), G(reen), and B(lue) channel, respectively. In order to suppress orientation information in isotropic brain regions, the 24-bit color value will be multiplied by FA. In the directionally encoded color map[9], red (R), green (G), and blue (B) colors are assigned to left-right, anterior-posterior, and superior-inferior orientations, respectively.

## DTI-Derived Images Provide High Contrasts to Delineate Neural Structures in Developmental Brains

Different neural structures in the fetal brains can be delineated with the DTI-derived images. Fig. 1 shows the DTI-derived images of a 21-week fetal brain. They are coronal images at the anterior commissure (ac) level. In Fig. 1a, the FA map characterizes the shapes of the diffusion tensor. Diffusion tensors at the dark regions are more like spheres and those at bright regions display more elongated shapes. Examples of diffusion tensors with both shapes are also illustrated in Fig. 1a. Some transient subcortical neural structures can be further differentiated based on FA in the developmental stage of the human fetus. For example, the ganglionic eminence (GE), a transient neural structure in the fetal brain, has high FA values and surrounds the low-FA caudate neucleus (Caud) around the ventricles (Fig. 1a). With the directionally encoded color map in Fig. 1b, most major white matter tracts, such as anterior commissure (ac), fornix (fx), cingulum (cg), and internal capsule (ic), can be clearly identified. The map of ADC, depicting the size of the diffusion ellipsoid, is shown in Fig. 1c. The ADC map displays very different contrasts from other

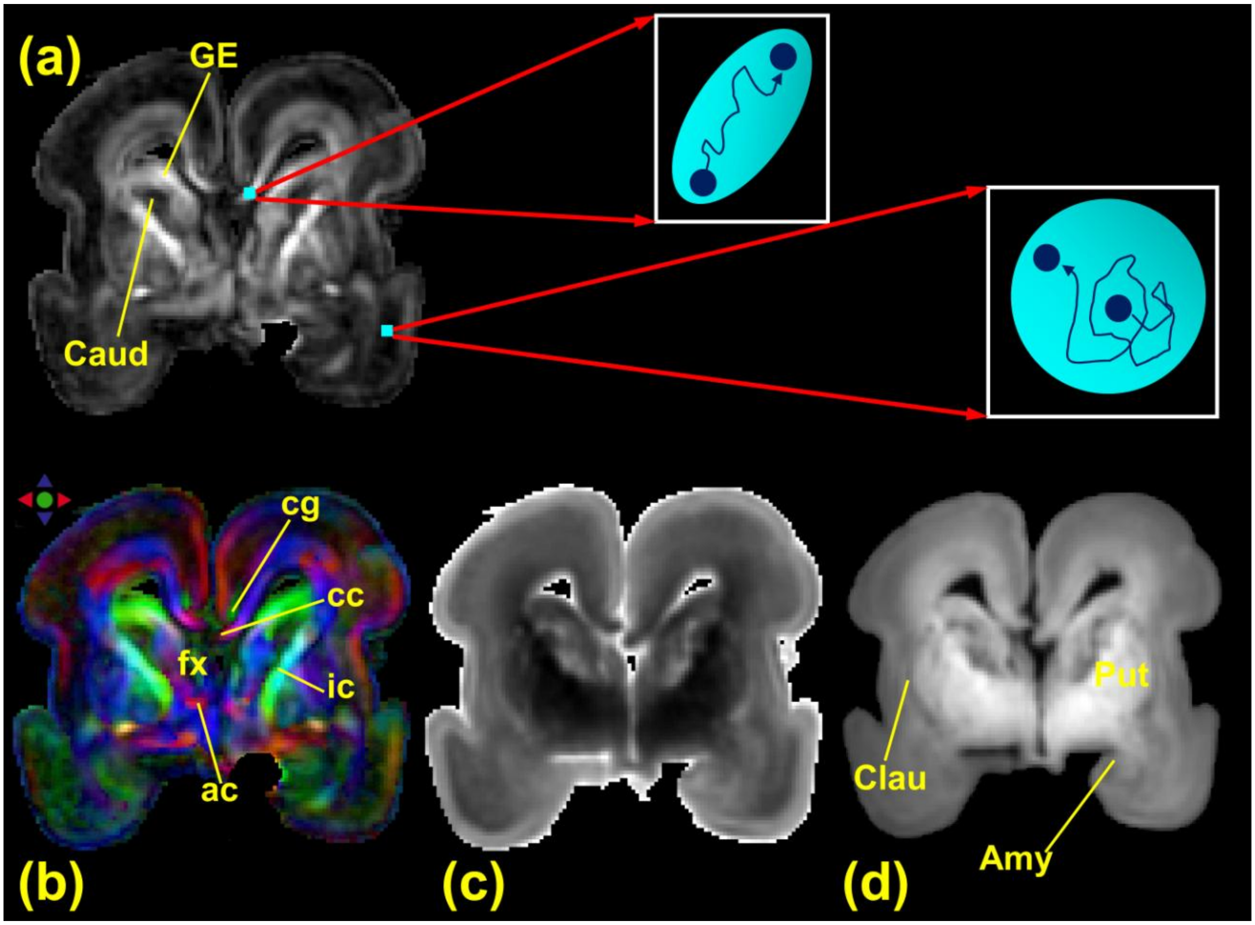

**FIGURE 1**. (a) FA map, (b) directionally encoded color map, (c) ADC map, and (d) aDWI, all derived from DTI and shown in a coronal slice of a 21-week fetal brain. High contrasts from DTI-derived images reveal many neural structures, including both white matter tracts and subcortical nuclei. The FA map in (a) describes the shape of the diffusion tensor, namely, how elongated a diffusion ellipsoid is. A voxel with high FA value indicates that it is characterized with a more elongated diffusion ellipsoid (left in the upper row), while a voxel with low FA value is characterized with the diffusion ellipsoid more like a sphere (right in the upper row). The blue small dots represent the water molecules and blue curves simulate the paths of the movement of water molecules. R(ed)/G(reen)/B(lue) in (b) indicate the left-right, anterior-posterior, and superior-inferior structural orientation, respectively. aDWI in (d) reveals the subcortical neural structures.

DTI-derived images. Fig. 1d is the averaged diffusion weighted image (aDWI) generated by summing up all DWIs. aDWI[21] has higher signal-to-noise-ratio (SNR) due to data averaging. Its contrast is similar to that of the DWI, which is acquired with the same diffusion gradient strength in all three directions. Other neural structures, such as claustrum (Clau), putamen (Put), and amagdala (Amy), can be delineated with this contrast. Detailed review of DTI and its derived metrics can be found in the literature[22,23,24].

## Primary Eigenvector of DTI can be used to Trace the Coherent Tracts and Reveal the Microstructures

The direction of the primary eigenvector (**$v_1$**) of the tensor is believed to align with the orientation of the underlying organized structures. This property makes the primary eigenvector useful to characterize the structures in two aspects, tracing the coherent tracts with tractography and revealing the microstructures.

Three-dimensional axonal bundles can be reconstructed from DTI data by tractography. DTI-based tractography refers to the techniques of connecting these primary eigenvectors to reconstruct the pathways of the coherent tracts. Streamline propagation methods[25,26,27,28,29,30,31] are mostly used.

DTI-based tractography requires a fractional anisotropy value higher than threshold and orientation continuity of primary eigenvector. Due to the fact that the diffusion tensor model oversimplifies the complex neural structures inside the brains, many other tractography algorithms that adapt more sophisticated diffusion models (e.g., [32,33,34,35,36,37,38]) rather than tensor have been postulated. However, for major white matter tracts, comparison of these DTI-based tractography and postmortem histological slides has shown that tractography based on DTI can reconstruct them accurately[12,39].

The second application of the measured primary eigenvector of the diffusion tensor is to reveal the microstructures in the cerebral wall. This is especially useful in developing brains. Unlike the adult brain, the cerebral wall in developing brains usually includes layers that have highly organized microstructures. These microstructures have been delineated in the developing brains of cat[40], mouse[16], rat[41], and human[42,43,44,45] with the primary eigenvector of the diffusion tensor. Because of the noninvasive nature of DTI, with 3D-DTI imaging technology, the orientations of these microstructures can be visualized and quantified in the three-dimensional space. For example, to represent the relative orientations of the microstructures in the cortical plate, the angles between the primary eigenvector (representing the orientations of the microstructures in the cortical plate) and normal vector of the cortical surface could be measured and visualized for the human fetal brains[45]. This measurement was used to indicate the distribution pattern of the orientations of the microstructures in the developmental brains.

## FETAL BRAIN DTI ATLAS

With the high contrasts of directionally encoded color maps, many white matter and subcortical neural structures can be identified. These structures can be further labeled with the guidance of the histological fetal brain atlas[46] and DTI white matter atlas of adult human[15] to generate the DTI atlas of the fetal brain. Details of the DTI-based atlas for the fetal brains in the second trimester can be found in the literature[45]. Here, sagittal-slice DTI fetal brain atlas is shown as an example.

In Fig. 2, sagittal slices of directionally encoded color maps are displayed to illustrate the development of commissural, limbic, and projection white matter tracts during development in the second trimester. Sagittal slices of aDWIs demonstrate the development of other major neural structures. From the midsagttial plane of the directionally encoded color map in Fig. 2, it is clear that a major commissural tract connecting two hemispheres, the corpus callosum, does not become apparent until 15 weeks. In a 13-week fetal brain, although the corpus callosum cannot be identified, many other commissural tracts, such as the middle cerebellar peduncle, optic chiasm, and anterior commissure, are observed. It should also be noted that the midsagittal corpus callosum of the fetal brain is extremely fragile and often severed (e.g., 19- and 21-week brains). In the parasagittal plane of the directionally encoded color map, the GE, which has a higher anisotropy than most subcortical nuclei[21], is well distinguished from others. The GE is a very large transient structure existing throughout the entire second trimester. The fornix (fx) and internal capsule (ic) are both well identified from the beginning to the end of the second trimester. They are also labeled in the parasagittal plane of the directionally encoded color map. Other major neural structures are labeled in the aDWI images in Fig. 2. Subcortical nuclei, such as putamen, caudate nucleus, and thalamus, are well observed in all second-trimester brains. The thickening of the cerebral wall and volume decrease of the ventricle during development in this period is well observed in the parasagittal aDWIs.

## THREE-DIMENSIONAL RECONSTRUCTION OF THE WHITE MATTER OF DEVELOPMENTAL BRAINS

Reconstruction of the white matter tracts directly delineates the three-dimensional morphology of these tracts. When applied to the same tracts of the developing brains, it can be used to characterize the developmental pattern of certain tracts. Fig. 3 shows the three-dimensional reconstruction of the corpus callosum and limbic tracts in a 19-week fetal brain, 0-year-old newborn brain, and 5-year-old child’s brain.

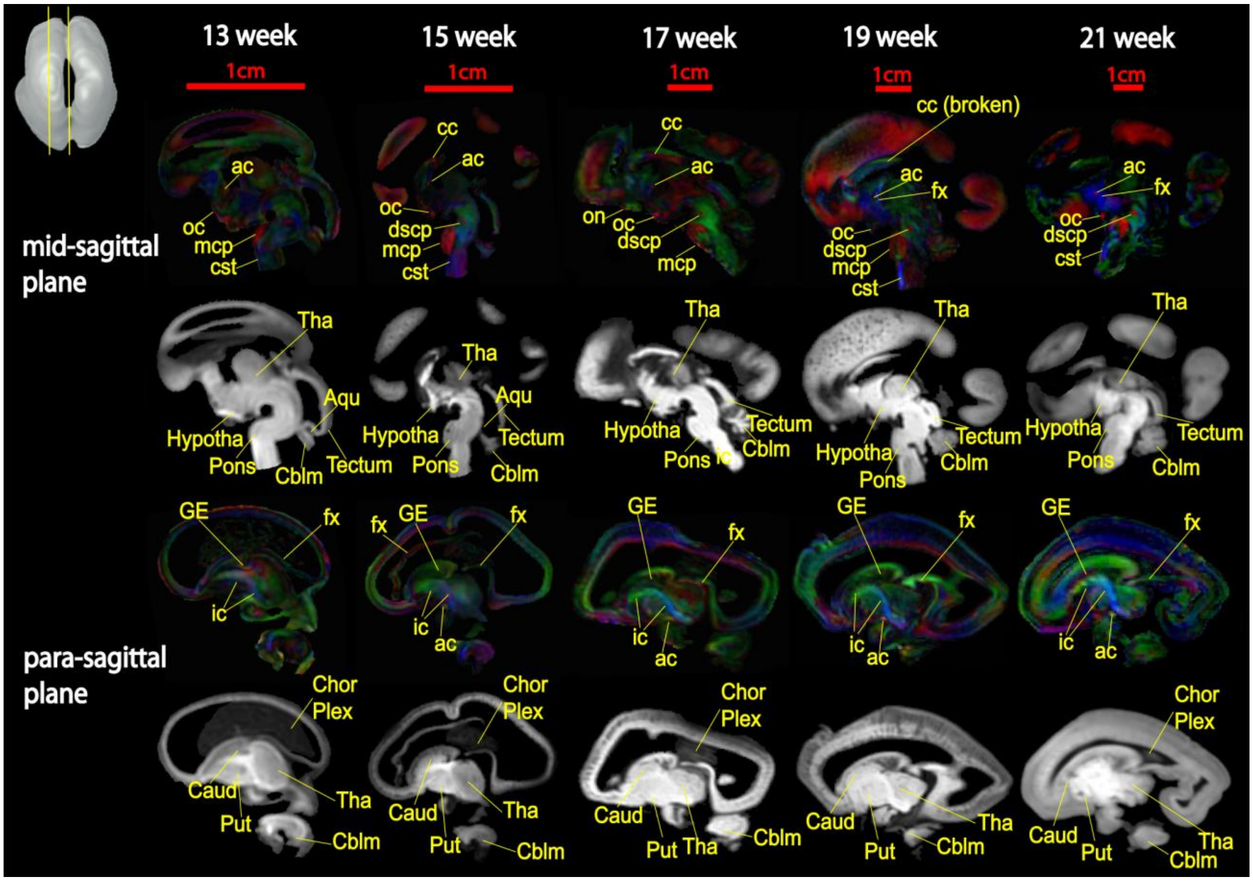

**FIGURE 2.** Fetal brain DTI atlas in sagittal planes. The location of each plane is demonstrated as yellow lines cross-sectioning the brain at the upper-left corner of each panel. Scale bars are shown at the top of the figure. Annotations of major white matter tracts and GE are shown in the directionally encoded color maps (the first and third rows) of fetal brains with 13, 15, 17, 19, and 21 weeks of gestational age. Annotations of other major neural structures are shown in the correspondent aDWI images in the second and fourth rows. Abbreviations not shown in text are as follows: cc, corpus callosum; cst, cortical spinal tract; dscp, decussation of superior cerebellar peduncle; mcp, middle cerebellar peduncle; oc, optical chiasm; on, optical nerve; Aqu, aquarium; Cblm, cerebellum; Chor Plex, choroid plexus; Hypotha, hypothalamus; Tha, thalamus.

It is clear from Fig. 3 that callosal tracts display the anterior-to-posterior developmental pattern, while the morphologies of limbic tracts are quite similar across the three developmental stages.

In the upper row of Fig. 3, it can be appreciated that formation of the corpus callosum is more advanced in the frontal lobe rather than the occipital lobe for the fetal brain at 19 gestational weeks. The forceps major (Fmajor) is much smaller than the forceps minor (Fminor) in the fetus. Callosal fibers in the occipital lobe are not well developed. This is also apparent in the midsagittal plane of the directionally encoded color maps in Fig. 2, in which the corpus callosum does not extend to the posterior region and the splenium is not yet well developed. For the 0-year-old newborn brain, the splenium and the forceps major are quite identifiable in Fig. 3, but the tracking results do not extend to some regions in the frontal and parietal lobe. These are the regions where anisotropy is relatively low even in adult brains (typically FA = 0.3 – 0.4), which is attributed to the mixture of three prominent tracts with different orientations (the corpus callosum, projection fibers, and the superior longitudinal fasciculus)[33]. The lower row of Fig. 3 shows three-dimensional reconstruction results of two important limbic fibers: cingulum (green fibers) and fornix (red fibers) connecting to the purple hippocampus. These are two of the most dominant tracts in the fetal brain and their entire trajectories are already developed at 19 gestational weeks. In addition, some association tract, such as superior longitudinal fasciculus (SLF), is completely missing in the brain of 19 gestational weeks.

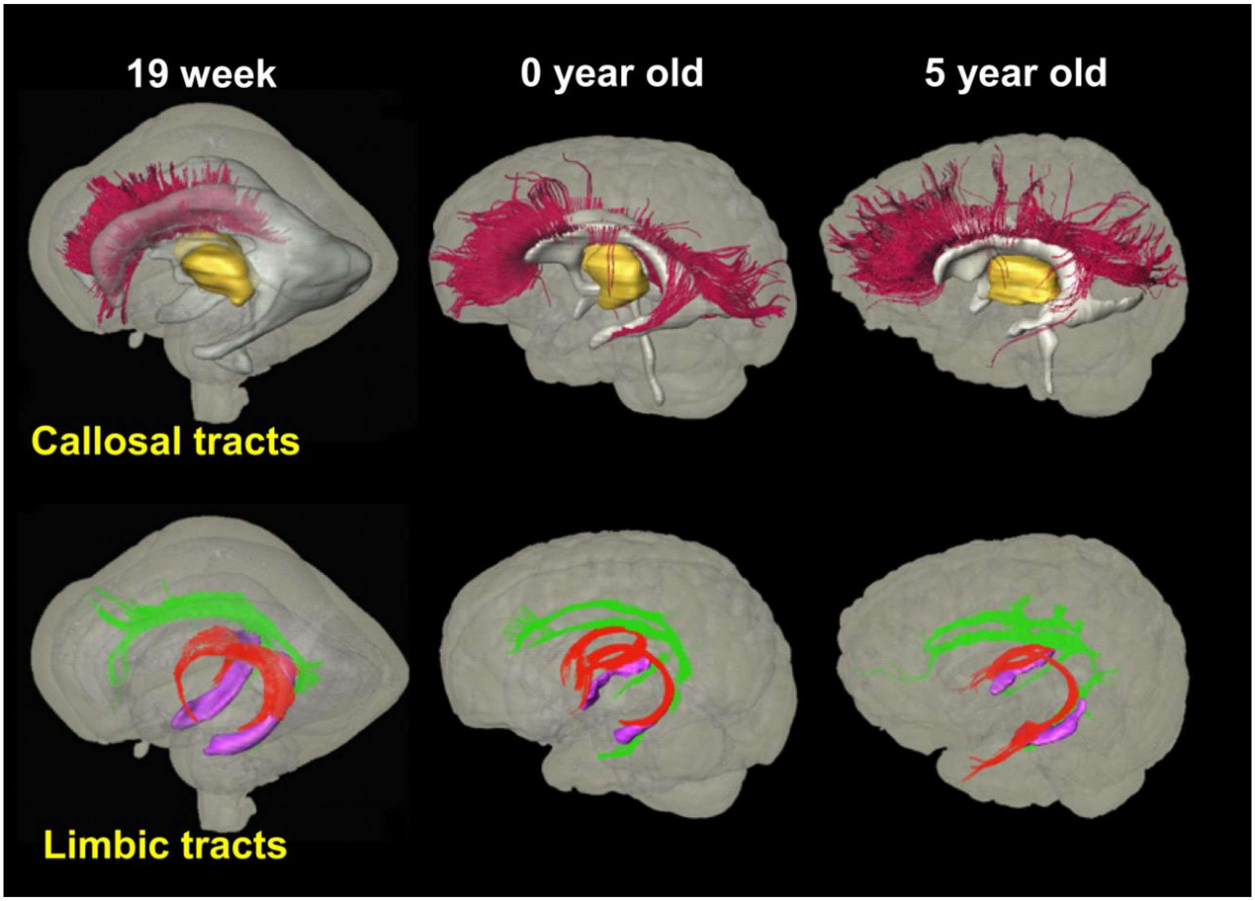

**FIGURE 3.** Upper row shows the three-dimensional tractography of corpus callosum for 19-gestational-week fetal brain, neonatal brain, and pediatric brain, respectively. The corpus callosum is not well developed in the posterior portion in the 19-week fetal brain. Lower row shows the three-dimensional reconstruction of the white matter tracts of the limbic system of the brain at the correspondent ages. In the lower row, the fornix (fx, red) and cingulum (cg, green) are reconstructed. The purple structure is the hippocampus.

## WELL-ORGANIZED MICROSTRUCTURES OF THE CEREBRAL WALL

DTI offers a unique insight into these microstructures and helps us to understand that development takes place in the cerebral wall in a well-ordered manner. The cerebral wall, which is the major component of the fetal brain, contains multiple layers[47] and undergoes active structural changes during fetal development. In the cerebral wall of the developing brain, both laminated and radial structures can be delineated with DTI. Histology and DTI are considered to be complementary to each other. Hence, Fig. 4 shows the axial image of the FA map and correspondent histological slide at the upper cerebrum of a 17-week fetal brain to illustrate the detailed structures in the developmental cerebral wall. From Fig. 4, both layered and radial structures can be clearly demonstrated with the FA map and the correspondent histological slide. Three layers can be identified in the FA image with one layer of lower FA nested in two layers of higher FA. The three layers identified by DTI in the cerebral wall are cortical plate, subplate, and inner layer from outer to inner direction[43,45]. The literature[45] has shown that the three layers are well identifiable for fetal brains from 15 to 22 gestational weeks of age, but not at 13 to 14 weeks. Primary eigenvectors from the diffusion tensor could be used to reveal the orientations of the microstructures in the cerebral wall. Among the three layers shown in Fig. 4, the outmost cortical plate has been found to have radial microstructures[42], which has been confirmed by several studies using *in vivo* DTI imaging of preterm newborn babies[43,44]. It has also been reported that the inner layer has

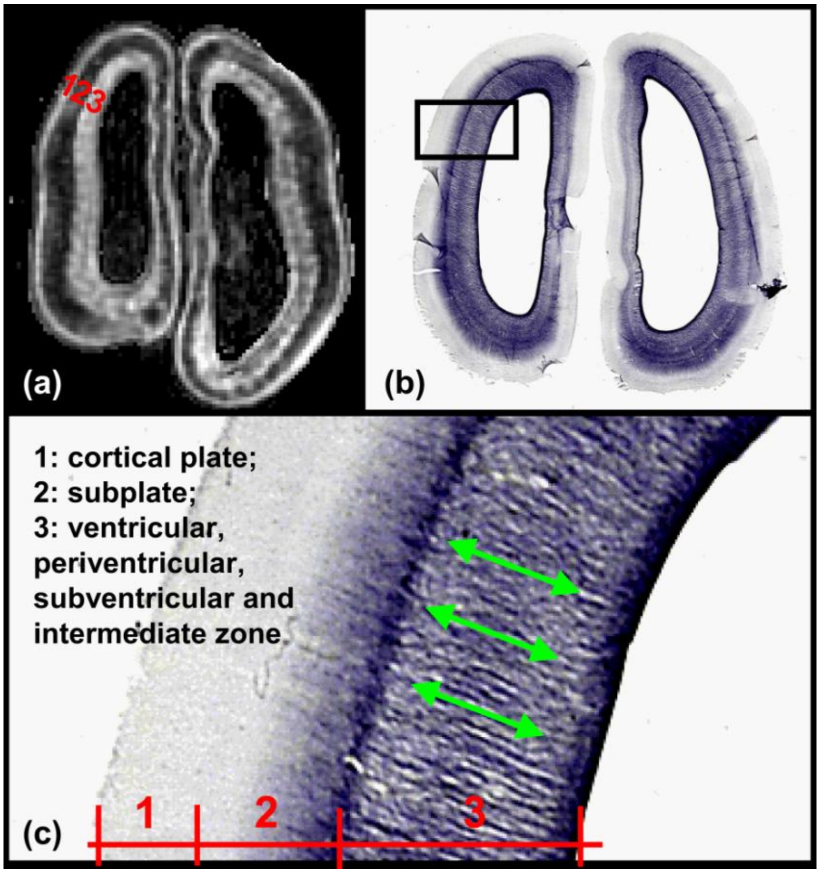

**FIGURE 4.** The axial slices of the FA map (a) and correspondent histological slide (b) of a 17-week fetal brain demonstrate laminated layers in the cerebral wall. Three layers with one layer of lower FA value nested in the two layers of higher FA are displayed in the FA map (a). These three layers are labeled with the red numbers. Enlargement of the cerebral wall in the black box of the histological slide (b) is shown in (c). The correspondent three layers can be identified and annotated in (c). Especially, the radial structures in the layer close to the ventricle are prominent. Green arrows in (c) indicate the orientations of these radial structures.

relatively higher FA than the middle subplate[43], which can be clearly observed in Fig. 4a. By enlarging part of the cerebral wall in the histological slide, it can be clearly observed that very organized radial structures are also dominant in the inner layer surrounding the ventricle (Fig. 4c).

Fig. 5a shows the aDWI and directionally encoded color map in an axial slice of the 21-week fetal brain. Fig. 5b,c shows the layers of the cerebral wall delineated by the aDWI and visualization of the primary eigenvector, respectively. The histological slide in Fig. 4 matches the visualization of primary eigenvectors in Fig. 5c, where the radial structures in the cerebral wall of a 21-week fetal brain are evident in layers 1 and 3 and absent in layer 2. Combining the results of Figs. 4 and 5 on structural characterization of the inner layer, it suggests that radial structure, rather than the tangential structure of fetal white matter (or intermediate zone) parallel to the ventricular surface and also residing in this layer, is dominant in the inner layer during the second trimester. FA of the inner layer during the second trimester is less than FA of the cortical plate, but higher than FA of the subplate. The inner layer contains several zones, namely ventricular, periventricular, subventricular, and intermediate zone, from inner to outer direction[47]. The interference of these radial and parallel structures may cause lower FA than that of the cortical plate where uniform radial structures were found.

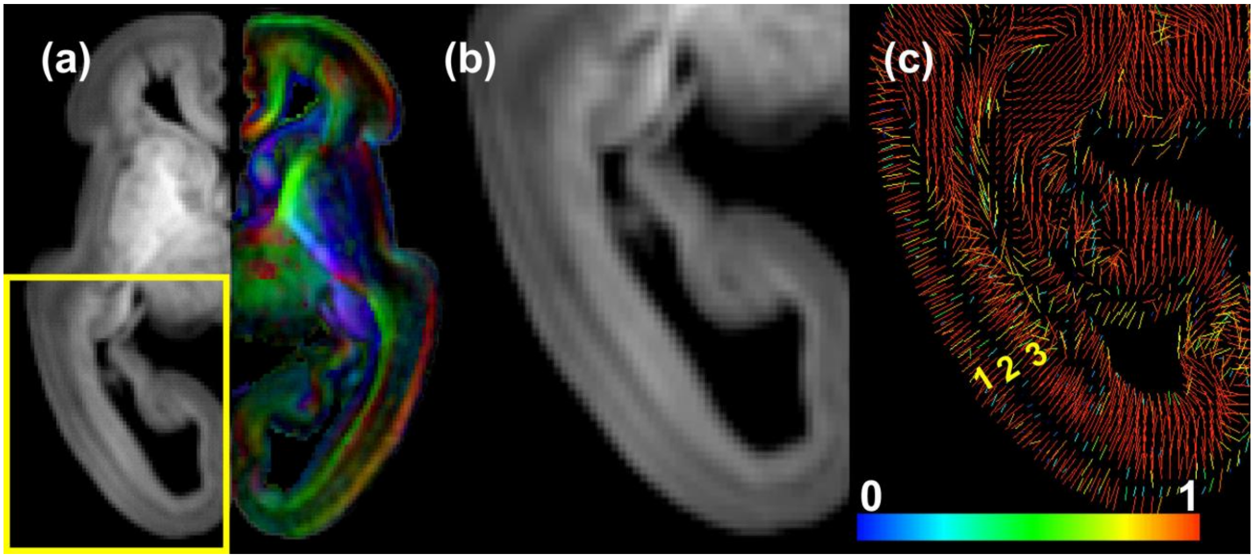

**FIGURE 5.** The axial averaged diffusion weighted image on the left and directionally encoded color map on the right of a 21-week brain are shown in (a). (b) is the enlarged yellow box in (a), showing the laminated layers in the cerebral wall and (c) is the visualization of the primary eigenvectors in the yellow box of (a). Three layers are labeled in (c) where primary eigenvectors of the diffusion tensor are oriented radially in layers 1 and 3. The black band between layers 1 and 3 in (c) is layer 2, where the primary eigenvectors are oriented in other direction, mostly perpendicular to the image slice. The color in (c) indicates the magnitude of the eigenvector projected to this axial plane, as illustrated by the color bar.

The radial structures existing in both cortical plate and inner layer, and revealed by both histology (Fig. 4c) and the primary eigenvector of DTI (Fig. 5c), might be formed by radially arranged scaffolding of the glial cells and are paths for neuron migration during development. They are highly regularized and perpendicular to the cerebral wall. The neurons are generated from close to the ventricle and migrate in the inner layer to the low-FA subplate zone where growing major afferent systems temporarily reside, establish synapses, and take part in cellular interactions that are crucial in subsequent cortical development. After neurons take a break from the migration at the subplate, they follow the radial direction in the cortical plate to the cortical surface. The whole migration process is from inside to outside across the cerebral wall. A previous study[45] indicates that these well-ordered radial structures in the inner layer exist from 15 to at least 22 weeks. Another study in the literature[42] found that the radial organization in the cortical plate disappears at 36 gestational weeks of age. These two findings[42,45] suggest that the radial organization of the cortical plate exists from 15 to 36 weeks.

## CONCLUSION

Application of DTI to the study of the structures of developmental brains has been described in several aspects. Neural structures of the developmental brains can be identified with high contrasts from DTI. It enables us to establish a DTI-based atlas. In addition, the primary eigenvectors of the diffusion tensor can be used to reconstruct the coherent tracts and reveal the microstructures in the cerebral wall. There are many transient structures that are unique in this developmental stage and disappear at term or during childhood development. These unique structures can be found in both white matter and the cerebral wall, and are usually associated with a specific neurodevelopment process. There are also neural structures existing in the adult human brain that are completely absent in the fetal brain. The DTI images, with their rich structural information, can bring us insight into the developmental pattern of neural structures. The atlas formed from the high-resolution DTI images of the fetal brain is also an invaluable reference for diagnostic radiology of preterm infants.

## ACKNOWLEDGMENT

The author would like to thank Dr. Susumu Mori for generous support of this study with his research resource. Studies presented in this review are supported by the National Institute of Health EB009545, AG20012, and RR15241.
